# Complex Implant-Prosthetic Rehabilitation Following Sports Trauma with 14 Years of Follow-Up: Case Report

**DOI:** 10.3390/dj9010006

**Published:** 2021-01-09

**Authors:** Alberto Murri dello Diago, Roberto Apponi, Vittorio Colombini, Lorenzo Mordini, Francesca Ideo

**Affiliations:** 1 Department of Dentistry and Oral Maxillofacial Surgery, University of Modena and Reggio Emilia, 41124 Modena, Italy; alberto.murridellodiago@gmail.com (A.M.d.D.); vittorio.colombini@hotmail.it (V.C.); 2 Department of Periodontology, School of Dental Medicine, Tufts University, Boston, MA 02111, USA; Lorenzo.mordini@tufts.edu; 3 Faculty of Dentistry, Oral & Craniofacial Sciences, King’s College of London, Guy’s Tower, Guy’s Hospital, Great Maze Pond, London SE1 1UL, UK; ideofrancesca@gmail.com

**Keywords:** dental trauma, tooth avulsion, luxation, implant-supported prosthesis, mini-implant

## Abstract

Tooth loss after traumatic dental injuries (TDI) often requires rehabilitation with a multidisciplinary treatment plan. In growing patients, the therapeutic approach may be different than in adults; the scientific literature offers alternative solutions even if they involve long, complex and uncomfortable treatments. Among the possible therapeutic options, implant-prosthetic treatment through the use of mini-implants is presented in this complex case report with a 14-year follow-up.

## 1. Introduction

Traumatic dental injuries (TDI) cause immediate damage in patients with a negative impact on quality of life [1]. Treatment plans are often multidisciplinary, lengthy and involve a team of dentists who must be able to solve various problems.

The difficulties in diagnosis are related to many aspects such as the patient’s age, the presence of deciduous and permanent teeth and bone stage development [2]. Surely, immediate treatment is important in the therapeutic management [3]. Therapeutic protocols must be chosen after careful evaluations [4]. Traumas involving the dental crown have current consolidated protocols; the continuous development of materials allows for conservative interventions that can restore and correct esthetics with reliable and minimally invasive techniques [5,6,7]. In case of traumas involving the dental pulp, the therapy will be more complex, even if official guidelines identify specific and predictable steps [8]. Nevertheless, in case of dental avulsion, the best therapeutic choice is tooth reimplantation, which can be influenced by many aspects such as root maturation stage or the extra-oral dry time.

When the treatment is delayed, guidelines are less specific and predictable. In these instances, it is necessary to evaluate which treatment is more suitable for the specific clinical scenario [9,10]. It is known that tooth reimplantation following dental avulsion is the first choice of treatment. The therapeutic approach may be complicated in growing patients. Tooth reimplantation can cause periodontal ligament inflammation, ankylosis or root resorption. An alternative, when reimplantation is not possible, is auto-transplantation [11]. It represents the most conservative therapy, and it allows bone and soft tissue growth. In the frontal area, it is possible to auto-transplant only premolar teeth with incomplete apex maturation [12]. Nevertheless, possible complications may arise, such as root resorption or non-stabilization of the reimplanted tooth [13]. After the healing of an auto-transplanted tooth, orthodontic and conservative treatment is necessary to have satisfactory esthetics.

In case of failure or when tooth replantation is not possible, the literature recommends other treatment options. One therapeutic alternative, widely discussed in the literature, is the closure of spaces through orthodontic treatment [14]. This option is related to the patient’s age and occlusion. When it is possible to use this technique, the tooth anatomy will be modified with direct or indirect techniques in order to achieve a harmonious and natural alignment. Another option is the replacement of avulsed teeth with fixed or removable prostheses, even if the limitations in growing patients are many [15].

A fourth therapeutic option is represented by implant-prosthetic rehabilitation. This choice is recommended in adult patients who have completed their skeletal growth. The continuous development of materials makes it possible to have specific implant fixtures, achieving ideal esthetics and eliminating mechanical problems, such as rotation of the prosthetic components [16,17]. Implant therapy in adolescents or growing patients has been widely evaluated, and some authors investigated and proposed the use of mini-implants to restore edentulous areas [18,19,20]. In similar cases, the possible therapeutic alternative is waiting for the growth end and, after, performing a regeneration of hard and soft tissues followed by traditional diameter implants placement. In the event that this therapeutic option is chosen, it will be necessary, for many years, to manage the lack of teeth lost in the trauma with fixed adhesive prostheses such as a Maryland bridge or with removable prostheses. The purpose of this case report is to provide an immediate therapeutic alternative with fixed implant-prosthetic rehabilitation supported by mini-implants in cases of traumatic loss of teeth in the esthetic area in the growing patient.

## 2. Case Report

**Figure 1:**
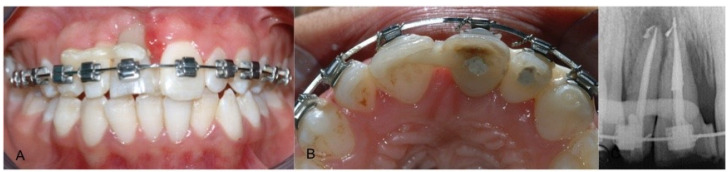
Intraoral pretreatment photographs and radiographs illustrating preoperative (**A**) buccal clinical view of anterior teeth, (**B**) occlusal clinical view of anterior teeth and (**C**) periapical X-ray.

A male patient, currently 27 years old, was seen for a consultation at the age of 13, fourteen days after a basketball game traumatic injury. The patient presented with an intrusive dislocation of the upper central and lateral incisor (#11, 12) which had been repositioned, treated endodontically and splinted with a multi-bracket orthodontic during an emergency visit. After some healing time, the splint was removed and upon clinical and radiographic evaluation, the failure of the reimplantation was noticeable (Figure 1).

Treatment was conducted in accordance with good clinical practice (GCP) established in the Declaration of Helsinki, and the treatment procedure, risks and benefits were completely explained to the parents and then written informed consent was obtained. After splint removal, the mobility of both teeth (degree 2 on the Miller scale) was evaluated and teeth #11 and #12 were extracted.

**Figure 2:**
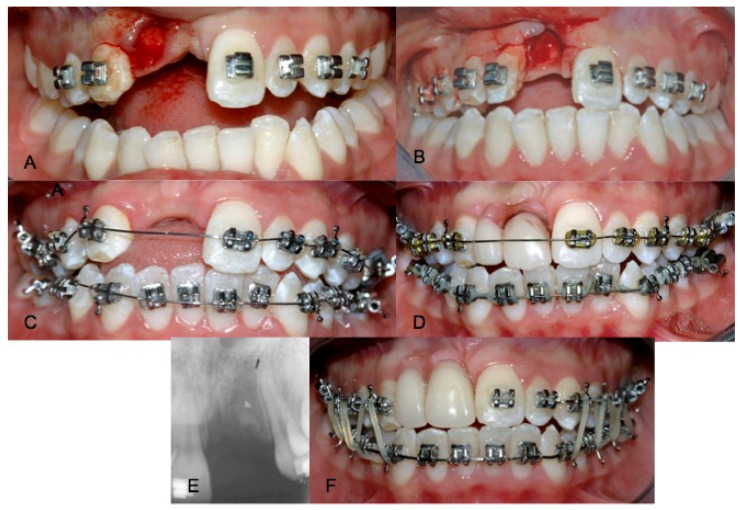
(**A**) Extraction of 12, (**B**) extraction of 11, (**C**) start of orthodontic therapy, (**D**) removable prosthesis placement, (**E**) periapical X-ray after two months and (**F**) orthodontic treatment after 4 months.

Orthodontic treatment was then performed on both arches to maintain spaces and harmonize the occlusion. A removable partial denture was delivered in order to restore the edentulous area (Figure 2).

**Figure 3:**
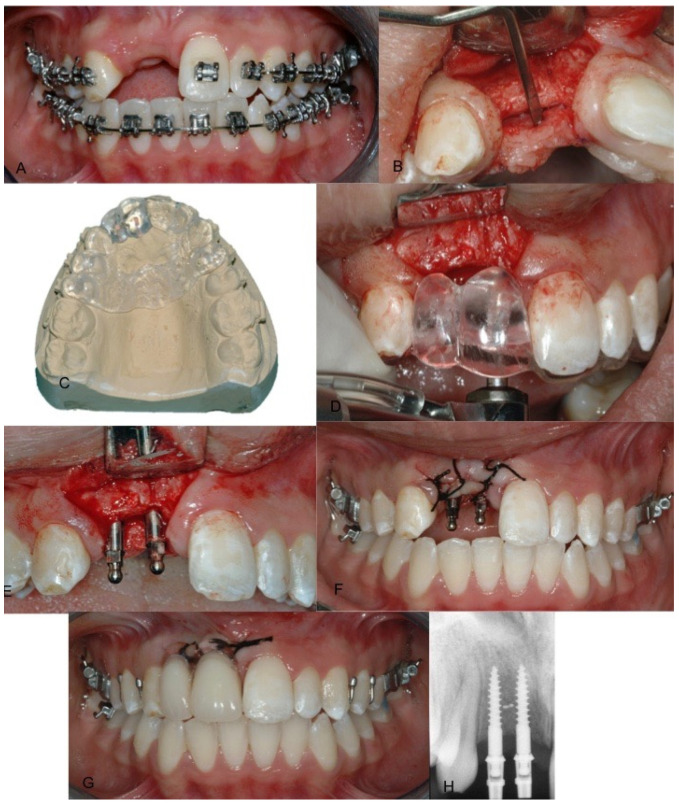
(**A**) Buccal clinical view after 4 months from extractions, (**B**) crestal bone volume, (**C**) surgical guide and upper model, (**D**) intraoral surgical guide placement, (**E**) paralleling during implant placement, (**F**) suture, (**G**) removable prosthesis placement and (**H**) immediate periapical X-ray control.

Four months later, intraoral and extraoral photographs, the impression and the facial arch for mounting the articulator were taken. Additionally, radiographic exams were required. After clinical and oral evaluations, two mini-implants were planned for the edentulous space via radiographic and stone casts evaluation. Under computerized local anesthesia, a full-thickness paramarginal incision was made. Bone was surgically exposed, and the surgical guide was positioned. Two 2.5 mm diameter by 13 mm length mini-implants (Sweden Martina, Italy) of grade 5 titanium alloy with a sandblasted surface were placed in areas #11 and #12 using a dynamometric torque wrench. The mini-implants were positioned through the use of a surgical guide. After the flap was primary closed around the prosthetic attachments, the removable prosthesis was repositioned, and radiographic verification was performed (Figure 3).

**Figure 4:**
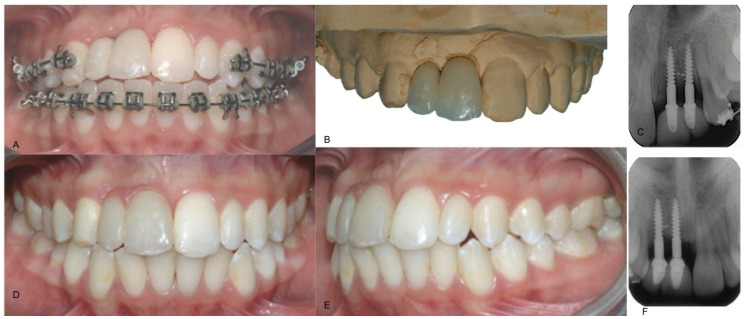
(**A**) Provisional resin crown on the implant, (**B**) plaster model of provisional crown, (**C**) periapical X-ray control after provisional crown placement, (**D**) frontal view of definitive crown after 13 years, (**E**) lateral view and (**F**) periapical X-ray control after 13 years.

After 14 days, a transfer was used to take the impression. Temporary resin crowns were placed, and orthodontic treatment was completed. Follow-ups were scheduled and after 6 months, orthodontic brackets were removed. After 3 months, clinical and radiographic examinations were performed and showed good healing of soft tissues without infection. Subsequently, temporary crowns were replaced with the final crowns. Final crowns included cervical pink porcelain contours, in order to improve the frontal esthetics (Figure 4). A multilayer individual EVA mouthguard was delivered to be worn during sport activities. After 14 years, the implants were stable and bone volumes were maintained. Occlusion was stable and from an aesthetic point of view, final crowns were well integrated with neighboring teeth and gingival tissues.

## 3. Discussion

Following tooth avulsion, it is necessary to make careful evaluations. The age of the patient and root maturation stage must certainly be taken into consideration before defining a treatment plan. The literature provides different therapeutic options, which all have positive and negative aspects. The treatment of choice for tooth avulsion is reimplantation, whenever and as soon as possible. If done within 60 min of loss, the success rates are high [21]. Auto-transplantation and orthodontic space closure are both reliable therapies even if not always indicated [22]. Decoronation can be used to maintain adequate tissue volumes by postponing therapies at the end of growth, when more definitive procedures can be performed [23]. In a case like the one presented, the literature presents two therapeutic options. The first is the maintenance of space followed by teeth auto-transplantation [11]. The second is orthodontic space closure [14] followed by the prosthetic treatment. Auto-transplantation in this case was not possible because the root development of the premolars was complete. The orthodontic closure of the spaces in this case in which the central incisor and the lateral incisor were missing would have led to a serious esthetic and functional defect. However, the treatment of avulsion teeth using implants has proved to be a very effective alternative [24]. The use of standard dental implants in growing patients has been extensively evaluated; the most common drawback is infra-occlusion [25]. The possibility of using mini-implants has proven to be effective. In fact, with the correct treatment planning, it is possible to resolve the esthetic and functional inconveniences caused by the loss of teeth. The management of the case through the years is generally not complicated. In fact, the current prosthetic techniques are able to adapt well to the gingival profiles even in growing patients. Another advantage of this technique is the possibility to remove mini-implants at the end of growth in case of infra-occlusion. The use of mini-implants in growing patients preserves bone tissue and restores function [26]. The authors have not experienced any case of infra-occlusion when treating patients with mini-implants. The result of this 14-year follow-up clinical case, associated with the promising data from the literature, allows the authors to conclude that mini-implants could be a valuable and reliable therapeutic option over time. All precautions should be taken during this treatment such as the possibility of limiting pain in anesthetic procedures using computerized systems [27]. Regular follow-up and use of dental protection systems such as mouthguards should be encouraged in patients playing sports since it would ensure protection from future traumas [28,29,30,31].

## 4. Conclusions

Traumatic loss of one or more tooth elements can cause serious esthetic and functional problems in patients of all ages. The treatment is often complex and involves a multidisciplinary approach. The prosthetic implant rehabilitation with mini-implants above presented proved to be predictable, effective, ready to be applied and easy to manage over the years. The possibility of carrying out a fixed rehabilitation compared to a removable one provides an evident benefit for patients’ quality of life. It also offers the possibility of performing corrective treatments when necessary. Compared to other therapeutic options, this technique was also found to be both less invasive and lengthy compared to standard dental procedures, reducing the treatment-related discomfort. Therefore, the possibility of using mini-implants for prosthetic rehabilitation following avulsion can be used as a valuable therapeutic alternative. The clinical aspects and the long follow-up of the case described contribute and confirm the possibility of using this technique with predictability. The technique is very innovative and therefore there are no long-term studies. The literature analysis on mini-implants used in growing patients shows an excellent survival rate. Randomized clinical trials are still needed to be able to further validate this technique.

## Data Availability

The data presented in this study are available in this article.
